# Antiatherogenic Effects of Quercetin in the THP-1 Macrophage Model *In Vitro*, With Insights Into Its Signaling Mechanisms Using *In Silico* Analysis

**DOI:** 10.3389/fphar.2021.698138

**Published:** 2021-07-27

**Authors:** Etimad A. Huwait, Salma Y. Saddeek, Rehab F. Al-Massabi, Sanaa J. Almowallad, Peter Natesan Pushparaj, Gauthaman Kalamegam

**Affiliations:** ^1^ Biochemistry Department, Faculty of Sciences, King Abdulaziz University, Jeddah, Saudi Arabia; ^2^ Cell Culture Unit and Experimental Biochemistry Unit, King Fahad Medical Research Centre, King Abdulaziz University, Jeddah, Saudi Arabia; ^3^ Chemistry Department, Faculty of Sciences, University of Hafr Al Batin, Hafr Al Batin, Saudi Arabia; ^4^ Biochemistry Department, Faculty of Sciences, University of Tabuk, Tabuk, Saudi Arabia; ^5^ Center of Excellence in Genomic Medicine Research, King Abdulaziz University, Jeddah, Saudi Arabia; ^6^ Department of Medical Laboratory Technology, Faculty of Applied Medical Sciences, King Abdulaziz University, Jeddah, Saudi Arabia; ^7^ Pharmaceutical Division, Nibblen Life Sciences Private Limited, Chennai, India

**Keywords:** THP-1 macrophages, inflammmation, stroke, brain destruction, cell migration, ingenuity pathway analysis, atherosclerosis, SwissTargetPrediction

## Abstract

**Background:** Atherosclerosis (AS), a major risk factor for stroke and brain tissue destruction, is an inflammatory disease of the blood vessels, and the underlying pathology is inflammation mediated by various chemokines and cytokines. Quercetin, a natural flavonol, is reported to have both anti-inflammatory and antioxidant properties. As such, in the present study, we evaluated the antiatherogenic effects of quercetin in a human THP-1 cell line *in vitro* and also the signaling mechanisms using *in silico* analysis.

**Materials and Methods:** THP-1 macrophages exposed to different concentrations of quercetin (5–100 μM for 24 h) were tested for cytotoxicity. Real-time gene expression assay for intercellular adhesion molecule-1 (ICAM-1) and monocyte chemoattractant protein-1 (MCP-1) was carried out following treatment with quercetin at 15 and 30 μM for 24 h either in the absence or presence of interferon (IFN-γ) for 3 h to induce inflammation. Monocyte migration and cholesterol efflux were also assessed.

**Results:** Quercetin did not exert any cytotoxic effects on THP-1 cells at the various concentrations tested. The gene expression assay showed a significant decrease in ICAM-1 (by 3.05 and 2.70) and MCP-1 (by 22.71 and 27.03), respectively. Quercetin at 15 µM decreased THP-1 monocyte migration by 33% compared to the MCP-1-treated cells. It also increased cholesterol efflux significantly by1.64-fold and 1.60-fold either alone or in combination with IFN-*γ*, respectively. Ingenuity Pathway Analysis of the molecular interactions of quercetin identified canonical pathways directly related to lipid uptake and cholesterol efflux. Furthermore, CD36, SR-A, and LXR-α also demonstrated significant increases by 72.16-, 149.10-, and 29.68-fold, respectively.

**Conclusion:** Our results from both *in vitro* and *in silico* studies identified that quercetin inhibited the THP-1 monocyte migration, MCP-1, and ICAM-1 and increased cholesterol efflux probably mediated *via* the LXR/RXR signaling pathway. Therefore, quercetin will help prevent cell infiltration in atherosclerotic plaques and reduce the risk of stroke or brain destruction.

## Introduction

Atherogenesis is a complex process that is driven by several factors, such as the oxidative process, vascular inflammation, lesion and plaque formation, instability, and vascular smooth muscle cell (VSMC) proliferation and migration (Yu et al., 2015). The early stage of atherosclerosis (AS) is characterized by low-density lipoprotein (LDL) accumulation, foam cell formation, and endothelial cell (EC) dysfunction, while in the late stage, smooth muscle cells infiltrate into the intima and eventually rupture the vessel wall (Kiaie et al., 2020). The inflammatory response to modified LDL (response to retention) and inflammatory response to damaged ECs (response to injury) are the two poorly understood pathways that lead to the development of AS (Burke and Howard, 2019). Once an inflammatory reaction in vessels ensues, ECs become damaged and atherosclerotic plaques are formed (Voloshyna et al., 2014). The plaque becomes stable due to the continuous release of the accumulated oxidized LDL (ox-LDL), a broad variety of chemokines and cytokines that recruit circulatory monocytes to ECs and other adhesion proteins to ECs (Ramji and Davies, 2015). During foam cell formation and subsequent disruption in the uptake and efflux of lipids, modified LDL particles are scavenged by two classes of scavenger receptors (SRs), cluster of differentiation 36 (CD36), and scavenger receptor class A member 1 (SR-A), which are expressed on the surface of macrophages. In contrast, liver X receptor alpha (LXR-α) is an essential regulator that contributes to the expression of ATP-binding cassette transporter A1 (ABCA1) and other transporters, such as apolipoproteins E and C1 (ApoE and ApoC1) (Hiebl et al., 2018; Jin et al., 2018). Monocyte chemoattractant protein 1 (MCP-1), also known as chemokine ligand 2 (CCL-2), is a member of the CC chemokine family and exhibits chemotactic activity for T lymphocytes, basophils, and monocytes (Deshmane et al., 2009). The key role of MCP-1 is to regulate the migration of monocytes into subendothelial spaces, which occurs when chemokine receptor type 2 (CCR2) interacts with MCP-1 (Ouchi et al., 2000; Deshmane et al., 2009). This chemokine also triggers adhesion molecules such as vascular adhesion molecule-1 (VCAM-1) and intercellular adhesion molecule-1 (ICAM-1), which are mediated by the binding of leukocyte integrins to ICAM-1 and VCAM-1, which are expressed by ECs (Blankenberg et al., 2003).

ICAM-1, also known as cluster of differentiation 54 (CD54) (Mruk et al., 2014), belongs to the immunoglobulin superfamily (Lim et al., 2015). ICAM-1 is continuously expressed at a low level but becomes increased following stimulation with cytokines such as IFN-γ or tumor necrosis factor-alpha (TNF-α) (Čejková et al., 2016; Lim et al., 2015; Jung et al., 2012). It is expressed in different types of cells, including macrophages, ECs, VSMCs, fibroblasts, and epithelial cells. ICAM-1 is associated with the migration of leukocytes to damaged tissues or the endothelium (Mruk et al., 2014). IFN-γ has been widely implicated as a proinflammatory cytokine in atherosclerosis and is considered a master regulator of atherosclerosis. (Moss et al., 2016; Gallagher et al., 2019). IFN-γ exerts its effects against infectious agents and contributes to host immune defense (Burke and Young 2019). However, as an inflammatory cytokine, its sustained inhibition is reported to be involved in tumor development by altering cellular and molecular mechanisms (Gotsman and Lichtman 2007).

Conventional therapeutics, including statins, do not offer complete protection or prevention of AS. Moreover, patients are susceptible to side effects such as myalgia and neurocognitive and ophthalmologic dysfunctions (Jung et al., 2012; Roy et al., 2020). Dietary modifications and lifestyle changes have been reported to have beneficial health effects. Therefore, a novel approach to prevent AS, such as the consumption of plants rich in flavanols, is being actively researched. Quercetin (Que) (3,5,7-trihydroxy-2-(3′,4′-dihydroxyphenyl)-4Hchromen-4-one) is a flavonol that is found abundantly in apples and onions (Wang et al., 2016). Quercetin has been reported to have potent antioxidant, anticancer, and antiviral activities (Wang et al., 2016; Dueñas et al., 2010; Rauf et al., 2018; Lim et al., 2015; Roy et al., 2020; Kim and Park, 2016). Earlier studies reported that quercetin activated the peroxisome proliferator activated receptor γ (PPARγ) and PPARγ–LXRα pathways and affected their target gene, ABCA1 (Lee et al., 2013; Sun et al., 2015). In the present study, we evaluated the *in vitro* anti-inflammatory and antiatherosclerotic effects of quercetin on THP-1 macrophages by studying cell viability, cell proliferation, inflammation-related gene expression (ICAM-1 and MCP-1), cholesterol efflux, and migration. In addition, the effects of quercetin on the molecular interactions, signaling pathways related to LXRα, cholesterol efflux and their target genes, and protein targets were evaluated using *in silico* analysis and SwissTargetPredictions.

## Materials and Methods

### Chemicals and Reagents

Quercetin (≥98% HPLC), Cat. No. 117-39-5, and IFN-γ (250 U/ml), Cat. No. 13265, were obtained from Sigma-Aldrich, Gillingham, United Kingdom. Dimethyl sulfoxide (DMSO; Cat. No. D12345) was obtained from Invitrogen (Carlsbad, CA, United States). The Pierce LDH cytotoxicity assay kit, Cat. No. 88954, was from Thermo Fisher Scientific (Waltham, MA, United States). One mM phorbol-12-myristate 13-acetate (PMA), Cat. No. J63916, was from Alfa Aesar (Thermo Fisher Scientific, United Kingdom). Roswell Park Memorial Institute (RPMI)–1640, Cat. No. A10491010; heat-inactivated fetal calf serum (HI-FCS), Cat. No. A3160502; 100 U/ml of penicillin and 100 μg/ml of streptomycin, Cat. No. 15140122; PBS, Cat. No. 10010015; MCP-1 (MCAF) Recombinant Human Protein, Cat. No. PHC1014; and (2 mmol/L) L-glutamine (200 mM), Cat. No. 25030081, were purchased from Gibco-BRL (Cheshire, United Kingdom). Total RNA was extracted using an RNeasy Mini kit (Cat. No. 74104; SYBR Green PCR kit, Cat. No. 204054, Qiagen, Manchester, United Kingdom). The ImProm-II™ Reverse Transcription System Kit (Cat. No. A3800) was purchased from Promega (Madison, WI, United States). The primers were purchased from Integrated DNA Technologies (Coralville, Iowa, United States). Falcon^®^ 12-well companion plates (Cat. No. 35334; SPL) were used for the monocyte migration test. The Cholesterol Efflux Assay Kit, Cat. No. ab196985, was from Abcam, United Kingdom.

### Preparation of Quercetin

Quercetin stock solution (1 M) was prepared using DMSO. The working stock was then prepared in complete media, and it was ensured that the final concentration of DMSO in culture was less than 1% to avoid toxicity to the cells.

### THP-1 Cell Culture

The human monocyte cell line THP-1 was received as a kind gift from the Molecular Biomedicine Unit at King Faisal Specialist Hospital and Research Centre in Riyadh, Saudi Arabia. THP-1 cells were cultured in tissue culture flasks (T-75) using an RPMI-1640 medium supplemented with 10% (v/v) heat-inactivated fetal calf serum (HI-FCS), 2 mmol/l L glutamine, and 1% antibiotic mixture (100 U/ml penicillin and 100 μg/ml streptomycin). The plated THP-1 cells were incubated in a CO_2_ incubator under standard culture conditions at 37°C with 5% CO_2_ in the atmosphere. Upon reaching confluence, the THP-1 cells were split every 2–3 days at a ratio of 1:5 to prevent overconfluence or to stop cell density exceeding 80%. Expanded cells were cryopreserved and stored in liquid nitrogen for subsequent use in the experiments.

### Lactate Dehydrogenase Assay

Briefly, THP-1 monocytes were seeded in 96-well plates at a density of 1 × 10^5^ cells/ml and differentiated into macrophages using 0.16 μg/ml (1 mM) PMA for 24 h. THP-1 macrophages were treated with 5, 10, 20, 30, 50, and 100 μM of quercetin for 24 h. Control cells were treated with the vehicle alone (DMSO). The LDH assay was performed according to the manufacturer’s protocol using 50 μl of the cell culture supernatant. Absorbance was measured at 460 nm using a spectrophotometer (BioTek Instruments, Winooski, VT, United States).

### Cell Proliferation Assay

Adherent macrophages from the abovementioned experiments were used for the cell proliferation assay. Briefly, the remaining 50 μl media were removed and replaced with 100 μl of crystal violet by dissolving 200 mg of crystal violet in 90 ml distilled water (w/v) and 10% ethanol (v/v) in each well. After 5 min of incubation at room temperature (RT), the cells were washed five times with 100 μl of 1x PBS 5 times. Then, 100 μl of the stop solution (0.1 M NaH_2_PO_4_) was added to the cells and the plate was gently agitated for 5 min on a shaker at 80 rpm. Absorbance was measured at 610 nm (BioTek Instruments, Winooski, VT, United States).

### Isolation of Ribonucleic Acid

THP-1 macrophages were seeded at a density of 1 × 10^6^/ml and induced with IFN-γ (250 U/ml) for 3 h to serve as an inflammatory stimulus. The cells were then treated with quercetin at 15 and 30 μM for 24 h. Control cells were treated with the vehicle alone (DMSO). Total ribonucleic acid (RNA) was extracted using the RNeasy Mini kit according to the manufacturer’s instructions. RNA quantity and quality were assessed using a Nanodrop™ (Wilmington, DW, United States).

### Affymetrix Microarray Processing

Transcriptional expression profiling was performed using Affymetrix GeneChip (Gene 1.0 ST, Affymetrix, Santa Clara, CA, United States) on the Affymetrix platform. This array is conceptually based on the human genome sequence assembly UCSC hg18, NCBI Build 36, and interrogated with a set of 764,885 probes and 28,869 annotated genes. It allows the researcher to profile the whole gene expression picture accurately and precisely as probes are based on the entire length of the gene. Target preparation, hybridization, washing, staining, and probe array scanning were performed to process the array.

### Circular Deoxyribonucleic Acid Synthesis

Double-stranded circular deoxyribonucleic acid (cDNA) was synthesized from extracted total RNA. An *in vitro* transcription reaction was performed to produce biotin-labeled cRNA, which was fragmented before hybridization.

### Circular Deoxyribonucleic Acid Hybridization

A hybridization cocktail was prepared with the fragmented target, labeled cRNA, control oligonucleotide B2, 20X eukaryotic hybridization, 2X hybridization mix, DMSO, and nuclease-free water. It was then hybridized with the GeneChip probe array at 45°C and 60 rpm for 16 h incubation.

### Fluid Station Setup and Affymetrix Array Washing and Staining

Experimental information such as probe array type, sample description, and comments was defined on a PC-compatible workstation using Affymetrix®Microarray Suite or GeneChip Operating Software (GCOS). The fluidics station was primed with appropriate buffers just before hybridization. The probe array underwent an automated washing and staining protocol at the fluidics station. The hybridized washed and stained probe arrays were kept at 4°C in the dark until scanning.

### Affymetrix Microarray Scanning

The Affymetrix® GeneChip® Scanner 3,000 was preheated for 15 min, and the arrays were maintained at room temperature before scanning. Affymetrix® Microarray Suite or GeneChip Operating Software (GCOS) defines the probe cells and computes the intensity for each cell, and a complete array image is saved with a data image file extension.

### Real-Time Quantitative Reverse Transcription-Polymerase Chain Reaction

Reverse transcription and cDNA synthesis were performed with random hexamers using the ImProm-IITM reverse transcription system kit. Real-time RT-PCR was performed with a Quanti-Fast SYBR Green PCR kit, using a StepOnePlus Real-time PCR machine (Applied Biosystems, Foster City, CA, United States). Primers for the following target genes ICAM-1 and MCP-1 were obtained from a previously published report (Moss et al., 2016): ABCA-1 (Moss, 2018), CD36, SR-A (Xu et al., 2006), and LXR-α (Saenz et al., 2018), and the housekeeping gene glyceraldehyde-3-phosphate dehydrogenase (GAPDH) was obtained from the work of Moss et al., 2016. Primer details are listed in Table 1. The PCR cycling conditions were as follows: preincubation, 120 s at 94°C; melting, 30 s at 95°C; annealing and extension, 60 s at 60 and 72°C, respectively; and melt curve, 10 s at 95 C. Relative gene expression was analyzed using the 2ΔΔ-CT method (Livak and Schmittgen, 2001; Rao et al., 2013).

**TABLE 1:** Details of genes and the specific primer sequences.

Gene	Primer sequence
ICAM-1	F: 5′-GAC​CAG​AGG​TTG​AAC​CCC​AC-3′
	R: 5′-GCG​CCG​GAA​AGC​TGT​AGA​T-3′
MCP-1	F: 5′-CGC​TCA​GCC​AGA​TGC​AAT​CAA​TG-3′
	R: 5′-ATG​GTC​TTG​AAG​ATC​ACA​GCT​TCT​TTG​G-3′
CD36	F: 5′-ATT GCC CTT TAC CTC GT-3′
	R: 5′-GCC TTG GAT GGA AGA ACA AA-3’
ABCA-1	F: 5′-AGT​GGA​AAC​AGT​TAA​TGA​CCA​G-3′
	R: 5′-GCA​GCT​GAC​ATG​TTT​GTC​TTC-3′
SR-A	F: 5′-ATT GCC CTT TAC CTC GT-3′
	R: 5′-TCA TTT CCT TTT CCC GTG AG-3′
LXR-α	F: 5′-AAG​CCC​TGC​ATG​CCT​ACG​T-3′
	R: 5′-TGC​AGA​CGC​AGT​GCA​AAC​A-3′
GAPDH	F: 5′-CTT​TTG​CGT​CGC​CAG​CCG​AG-3′
	R: 5′-GCC​CAA​TAC​GAC​CAA​ATC​CGT​TGA​CT-3′

### Monocyte Migration Assay

The migration of THP-I monocytes was tested using Falcon ® 12-well companion plates with the top half apical compartments of Falcon cell culture inserts (8 μM pore size). The chemokine MCP-1 (20 ng/ml) was added to the bottom wells to act as a chemoattractant. THP-I monocytes (5 × 10^5^ cells/ml) were plated in the top wells and treated with quercetin (15 μM). Control wells were treated with the vehicle alone (DMSO). The modified Boyden chambers were then incubated for 3 h at 37°C in 5% (v/v) CO_2_ (J. W. E. Moss et al., 2016).

The THP-1 monocytes that had migrated to the bottom wells through the pore and those that were attached to the underside of the membrane were collected by washing with 0.5 ml of PBS (1x, pH 7.4). The cells in the bottom well were collected and centrifuged (250 × *g* for 5 min), and the cell pellet was resuspended in 1 ml fresh media and counted using a hemocytometer (Moss et al., 2016).

### Cholesterol Efflux Assay

Cholesterol efflux assay was performed using the commercial Cholesterol Efflux Assay kit as previously described (Almowallad et al., 2020; Almassabi et al., 2021) to analyze the impact of quercetin on AS progression in our cellular model. THP-1 monocytes were seeded at a density of 1 × 10^5^ cells/well in the culture medium (100 μl media/well) in a 96-well black plate with a clear bottom (kit content) and incubated for 1 h to settle down. Then, to enable differentiation into adherent macrophages, 0.16 μg/ml (1 mM) of PMA was added and cells were incubated overnight. To initiate the development of foam cells, 250 U/ml IFN-γ was added to one sample set and the vehicle was incubated for 3 h. Cell monolayers were washed with serum-free media after incubation. Just before using the labeling reagent and equilibration buffer (kit contents), 100 μl/well was added. The labeling reagent was removed after overnight incubation, and the cells were gently washed with 200 μl serum-free media. For both sets of samples, 100 μl of fresh RPMI medium containing 15 μM quercetin was added and incubated at 37° in an incubator containing 5% CO_2_ for 24 h. The positive control (20 μl, kit content) was added to the respective wells, and only the serum-free medium was added to the negative control wells. The cells were incubated in a 37°C incubator containing 5% CO_2_ for another 4 h. Then, the supernatant was transferred to a new 96-well plate and the cell monolayer was solubilized with 100 μl of cell lysis buffer by gentle agitation on a plate shaker for 30 min at RT. An Ex/Em of 482/515 nm was used to test the fluorescence of the two plates. Cholesterol efflux was determined by dividing the fluorescence intensity of the medium by the total fluorescence intensity of the medium and the cell lysate, and the cholesterol efflux percentage was determined.

$$\text{\%}\;cholesterol\;Efflux=\frac{\text{Fluorescence Intensity of Med}}{\text{Fluorescence Intensity of Media }+\text{ Cell Lysate }}\times \;100.$$

### Ingenuity Pathway Analysis

Ingenuity Pathway Analysis (IPA) software (Qiagen, United States) was used to identify the associated canonical pathways with quercetin (Bahlas et al., 2020; Jafri et al., 2020). The molecules were then used in the expression analysis to predict signaling mechanisms, targets, and their association with quercetin using direct or indirect relationships. Network predictions based on the input of molecules were generated using algorithms contained in the Ingenuity Knowledge Base (Bahlas et al., 2020). The percentage and number of uploaded genes/molecules matching the genes of a canonical pathway were measured as Z-score, ratio, or Fisher’s exact test for significance (Jafri et al., 2020).

### SwissTargetPrediction

To identify the beneficial effects of quercetin in relation to AS, target prediction was performed using the SwissTargetPrediction web tool with an update on bioactivity data and retrained and redefined similarity thresholds (Daina et al., 2019; Bahlas et al., 2020). Ligand-based target prediction was performed based on the similarity between the query molecule and the compiled curated collection using 2D and 3D similarity measures within the larger bioactivity data of ChEMBL version 23. A combined score of higher than 0.5 indicates that the molecules share a common protein target (Daina et al., 2019; Bahlas et al., 2020). In reverse screening, the combined score helps to calculate the probability of targeting a given protein. Dual-based reverse screening has demonstrated high performance in predicting macromolecular targets (Kalamegam et al., 2020).

### Statistical Analysis

Data were analyzed using the statistical program GraphPad Prism 8 (GraphPad Software Inc. United States). Analysis of variance (ANOVA) was performed followed by Tukey’s or Dunnett’s multiple comparisons test, and results were expressed as mean ± standard deviation (SD). Results were obtained after three individual experiments, and statistical significance was assigned for a *p* value ≤0.05.

## Results

### Lactate Dehydrogenase Assay and Crystal Violet Assay

**FIGURE 1:**
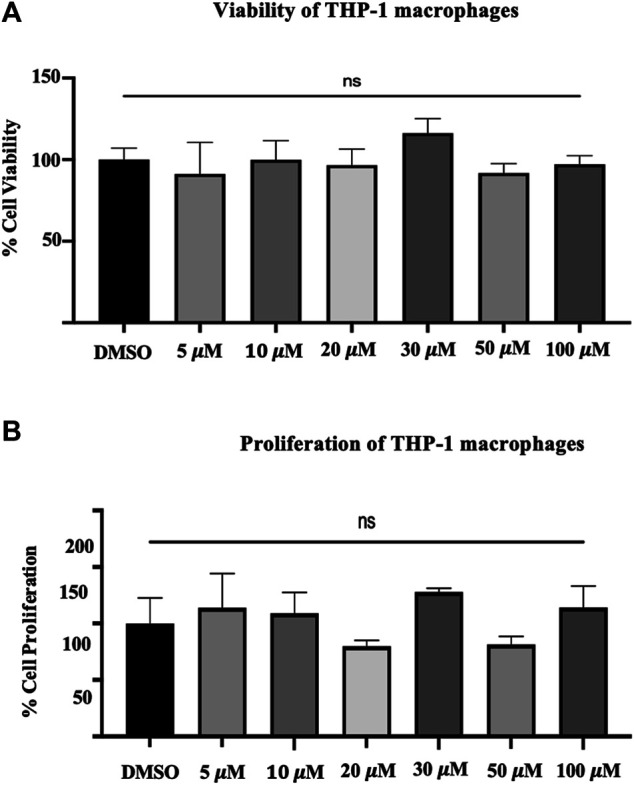
**(A)** The viability of THP-1 macrophages. The lactate dehydrogenase assay on THP-1 macrophages following treatment with different concentrations (5–100 μM) of quercetin for 24 h. **(B)** Crystal violet assay was carried out with the remaining attached macrophages from the previous experiment of the LDH assay. The values are expressed as mean ± SD of three independent experiments. Statistical analysis was conducted using a one-way ANOVA with Dunnett's multiple comparisons test on log-transformed data where *p < 0.05, **p < 0.01, ***p < 0.001, and ****p < 0.0001.

The LDH assay indicates loss of cell membrane integrity and, hence, reflects cell viability. The LDH assay performed on THP-1 macrophages generally showed a decrease with the different concentrations of quercetin (5–100 μM) compared to the control (Figure 1A). The mean fold changes were 8.9%, 0.19%, 3.47%, 16.1, 2.84, and 8.31% for the concentrations at 5–100 μM, respectively, and these fold changes were not statistically significant compared to the control (*p* = 0.7199) (Figure 1A).

Cell proliferation was measured by crystal violet assay; likewise, the mean fold changes were 13.96, 8.98, 20.11, 27.84, 18.50, and 14.24% for the concentrations from 5 to 100, respectively (Figure 1B). The observed fold changes were not statistically significant compared to the control (*p* = 0.0.0662) (Figure 1B).

### ICAM-1 and MCP-1 Gene Expression in Quercetin-Treated THP-1 Macrophages

**FIGURE 2:**
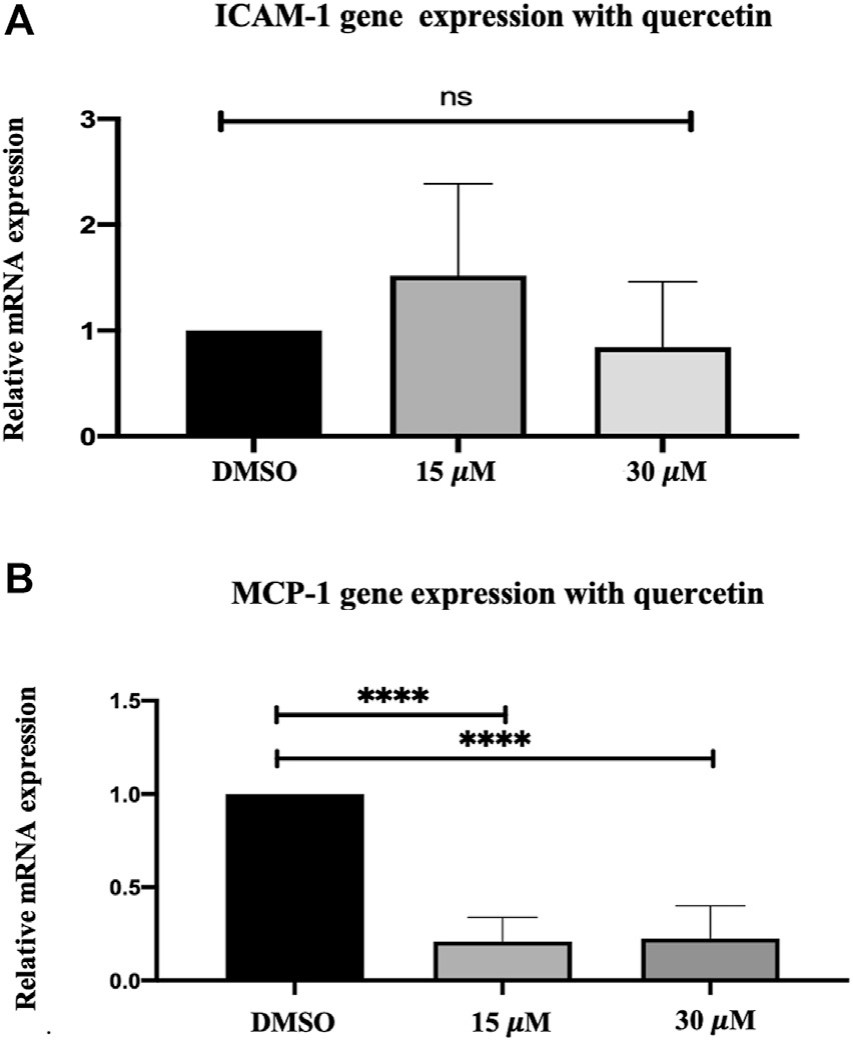
Gene expression assay in THP-1 macrophages following quercetin treatment. Real-time gene expression: The effect of quercetin in THP-1 monocytes following stimulation with PMA on the expression of **(A)** ICAM-1 and **(B)** MCP-1 genes. The basal gene transcript levels of ICAM-1 and MCP-1 were assessed following treatment with quercetin at two different concentrations. The data are presented as the mean ± SD. Statistical analysis was conducted using a one-way ANOVA with Dunnett's multiple comparisons test on log-transformed data where *p < 0.05, **p < 0.01, ***p < 0.001, and ****p < 0.0001.

Treatment of THP-1 macrophages with quercetin at 15 and 30 μM concentrations for 24 h demonstrated a biphasic response with ICAM-1 and decreased expression of MCP-1 compared to the control. ICAM-1 showed an increase of 0.52-fold at 15 μM and a decrease of 0.16-fold at 30 μM. Both the increase and decrease observed with ICAM-1 were not statistically significant (*p* = 0.3447 and *p* = 0.90940, respectively) (Figure 2A). MCP-1 demonstrated a decrease in gene expression at both 15 and 30 μM concentrations. The decrease by 0.79-fold at 15 μM and 0.78-fold at 30 μM was statistically significant compared to the control (*p* < 0.0001) (Figure 2B).

### Quercetin Reduces IFN-γ-Induced mRNA Overexpression of ICAM-1and MCP-1 in THP-1 in Macrophages

**FIGURE 3:**
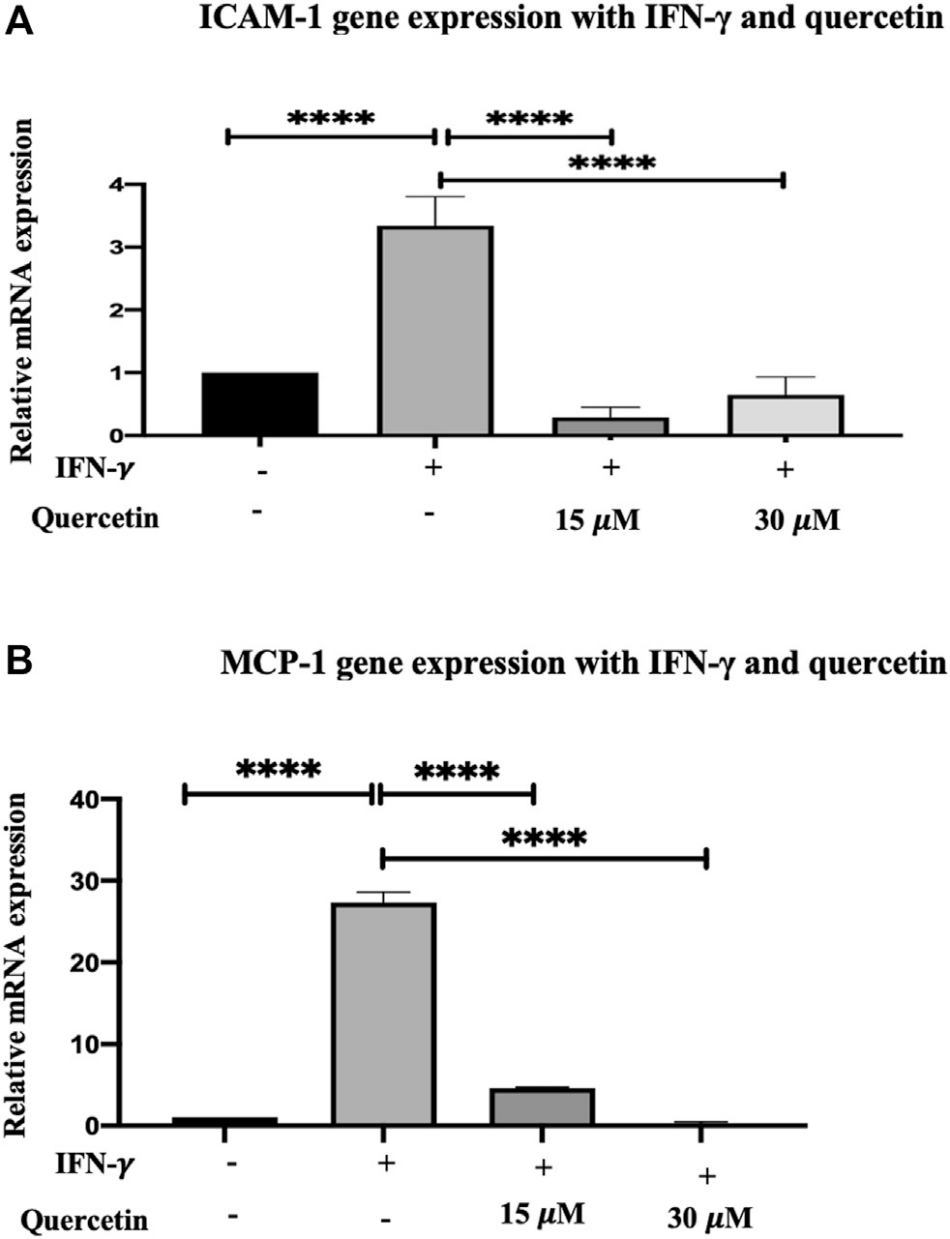
Gene expression assay in THP-1 macrophages induced with IFN-γ and treatment with quercetin: the effect of quercetin on the expression of **(A)** ICAM-1 and **(B)** MCP-1 showing their genes in THP-1 macrophages. The THP-1 monocytes were earlier differentiated into macrophages using PMA, and the gene transcript level of ICAM-1 and MCP-1 was assessed in the presence of IFN-γ (250 U/ml) induction for 3 h and then treated with two concentrations of quercetin for 24 h. The data are presented as the mean ± SD. Statistical analysis was conducted using a one-way ANOVA with Tukey’s multiple comparisons test on log-transformed data where *p < 0.05, **p < 0.01, ***p < 0.001, and ****p < 0.0001.

Adhesion molecules and chemokines tend to be part of the inflammatory response, and their expression is induced by IFN-γ. Treatment of THP-1 macrophages with IFN-γ (250U/ml) for 3 h increased the expression of both ICAM-1 and MCP-1 genes. The increase in ICAM-1 by 2.34-fold and MCP-1 by 26.33-fold were statistically significant compared to the control (*p* < 0.0001 and *p* < 0.0001, respectively) (Figure 3A and Figure 3B).

Treatment of THP-1 macrophages with quercetin (15 and 30 μM) following initial IFN-γ stimulation demonstrated a decrease in ICAM-1 by 3.052-fold (*p* < 0.0001) and 2.70-fold (*p* < 0.0001) compared to IFN-γ. Likewise, following initial stimulation with IFN-γ, treatment with quercetin at 15 and 30 μM demonstrated decreases in MCP-1 by 22.71-fold (*p* < 0.0001) and 27.03-fold (*p* < 0.0001), respectively.

### Quercetin Inhibits Monocyte Migration Toward MCP-1

MCP-1 is considered a marker for the early stage of AS progression and plays a significant role in the recruitment and migration of monocytes into the subendothelial spaces. In response to stimulation with MCP-1 (20 ng/ml), there was an increase in the migration of THP-1 monocytes by 51.75% compared to the control, and this increase in migration was statistically significant (*p* < 0٫0,001).

**FIGURE 4:**
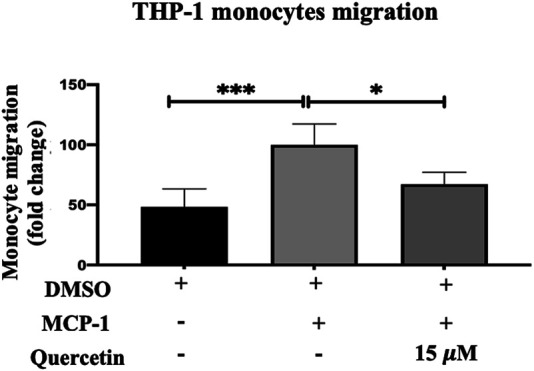
THP-1 monocyte migration. The migration of THP-1 monocytes was evaluated following stimulation with MCP-1 (20 ng/ml) alone or with MCP-1 (20 ng/ml) in the presence of quercetin (15 μM) for 3 h. The data are presented as the mean ± SD from three independent experiments. Statistical analysis was performed using a one-way ANOVA with Tukey’s multiple comparisons test where *p < 0.05, **p < 0.01, ***p < 0.001, and ****p < 0.0001.

THP-1 monocyte migration following treatment with 15 μM quercetin demonstrated a mild decrease of 32.86% compared to the MCP-1 treated only, and this decrease was statistically significant (*p* < 0.0158) (Figure 4).

### Quercetin Increases the Cholesterol Efflux in THP-1 Macrophages

**FIGURE 5:**
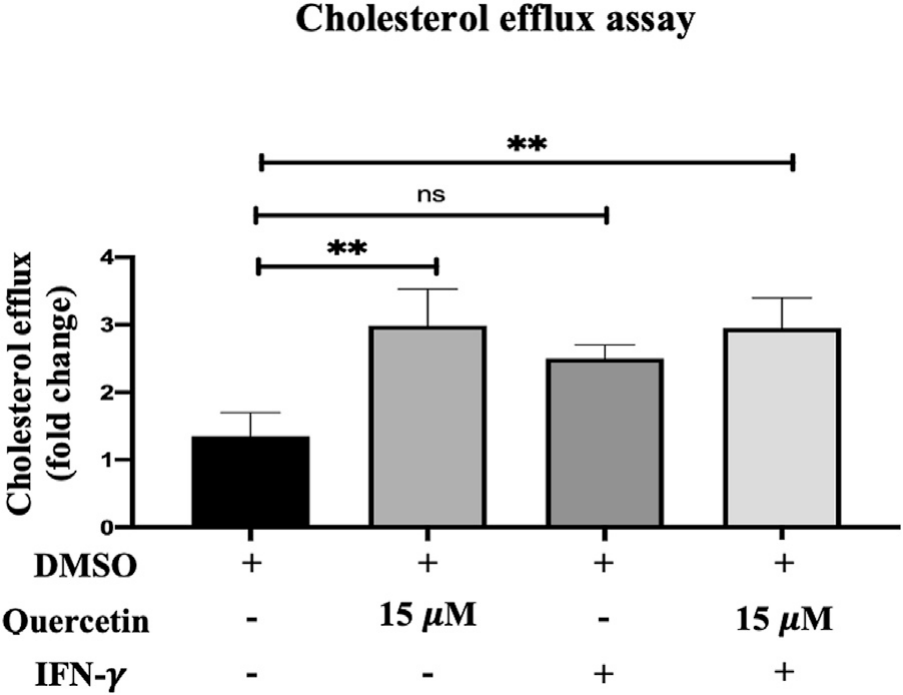
Cholesterol efflux assay. The effect of quercetin on cholesterol efflux in the presence or absence of IFN-γ-induced THP-1 macrophages is shown. The data are presented as the mean ± SD from three independent experiments. Statistical analysis was performed using a one-way ANOVA with Tukey’s multiple comparisons test where *p < 0.05, **p < 0.01, ***p < 0.001, and ****p < 0.0001.

Cellular cholesterol efflux is another key step in AS progression. The cholesterol efflux evaluated in THP-1 macrophages was increased following treatment with quercetin (15 μM) for 24 h, IFN-γ (250U/ml) for 3 h, and with the combination of IFN-γ and quercetin (Figure 5). The increase in cholesterol efflux was 1.6-fold, 1.1-fold, and 1.6-fold with Que, IFN-γ, and their combination, respectively. However, statistically significant increases in cholesterol efflux were observed only with quercetin (*p* < 0.0071), and the combination of quercetin and IFN-γ (*p* < 0.0055) was statistically significant compared to the control.

### SwissTargetPrediction

**FIGURE 6:**
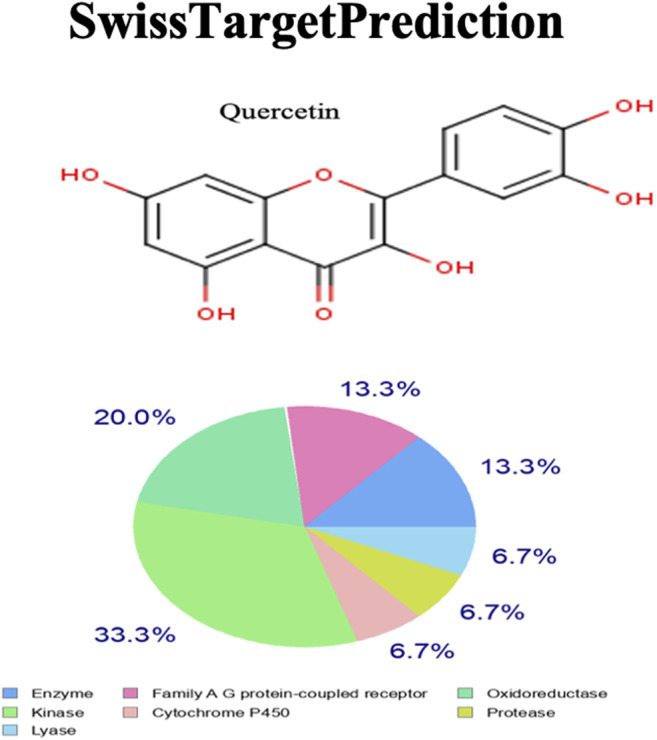
SwissTargetPrediction showing the top biomolecular targets for quercetin.

Quercetin targets the following molecular/biochemical pathways: G-protein coupled receptor, oxidoreductase, protease, cytochrome P450, kinase, and lyase. Although more bioactive targets were curated based on 2D and 3D similarity measures, the abovementioned targets were among the top 15 curated based on our query molecule (Figure 6). Most of the protein targets identified using the latest SwissTargetPrediction (2019 version) have high probability values (Figure 6) and are potential therapeutic targets for AS.

### Differential Gene Expression in Differentiated THP-1

Comparison of the genome-wide expression after 24 h exposure of THP-1 cells to quercetin at 15 μM compared to the untreated control cells revealed 27 DEGs. Fifteen genes were upregulated (Table 2), and 12 genes were downregulated (Table 3), with a cutoff of *p* value <0.05 and fold change more than ±2.

**TABLE 2:** Top 15 significantly upregulated genes (treatment vs. control).

Gene assignment	Gene symbol	*p* value	Fold change (case vs. control)
NM_000072//CD36//CD36 molecule (thrombospondin receptor)//7q11.2//948///	CD36	0.00346,366	12.4453
NM_002445//MSR1//macrophage scavenger receptor 1//8p22//4,481///NM_138,715//M	MSR1	0.0426,456	11.0764
NM_001,130,101//NR1H3//nuclear receptor subfamily 1, group H, member 3//11p11.2//	NR1H3	0.00101,781	7.75181
NM_001115//ADCY8//adenylate cyclase 8 (brain)//8q24//114///XM_005,250,769//AD	ADCY8	0.000455,717	5.30974
NM_002204//ITGA3//integrin alpha 3//17q21.33//3675///XM_005257308//ITGA3//	ITGA3	0.00139,344	5.00455
NM_001256324//CACNA1G//calcium channel, voltage-dependent, T type, alpha 1G subunit	CACNA1G	0.00580,794	4.69458
NM_000041//APOE//apolipoprotein E//19q13.2//348///NM_001302688//APOE//apo	APOE	0.031144	4.47527
NM_001645//APOC1//apolipoprotein C-I//19q13.2//341///XM_005258855//APOC1//	APOC1	0.0263,444	4.47513
NM_001008540//CXCR4//chemokine (C-X-C motif) receptor 4//2q21//7852///NM_0,034	CXCR4	0.0298,972	4.14413
NM_006206//PDGFRA//platelet-derived growth factor receptor, alpha polypeptide//4q	PDGFRA	0.0305,225	3.24131
NM_002203//ITGA2//integrin, alpha 2 (CD49B, alpha 2 subunit of VLA-2 receptor)//5	ITGA2	0.00438,663	3.19315
NM_002185//IL7R//interleukin 7 receptor//5p13//3575///NR_120,485//IL7R//in	IL7R	0.0233,437	3.11418
NM_003263//TLR1//toll-like receptor 1//4p14//7096///XM_005262662//TLR1//t	TLR1	0.00955,191	3.05925
NM_002211//ITGB1//integrin beta 1//10p11.2//3,688///NM_033668//ITGB1//inte	ITGB1	0.0338,444	2.18716
NM_000633//BCL2//B-cell CLL/lymphoma 2//18q21.3//596///NM_000657//BCL2//B	BCL2	0.00533,366	2.01142

**TABLE 3:** Top 12 significantly downregulated genes (treatment vs. control).

Gene assignment	Gene symbol	*p* value	Fold change (treatment vs. control)
NM_001204406//ALOX5AP//arachidonate 5-lipoxygenase-activating protein//13q12//2	ALOX5AP	0.00470,171	−5.72453
NM_000104//CYP1B1//cytochrome P450, family 1, subfamily B, polypeptide 1//2p22.2	CYP1B1	0.0114,268	−5.3173
NR_002324//SNORA62//small nucleolar RNA, H/ACA box 62//3p22.1//6,044///ENST000	SNORA62	0.0136,371	−4.23037
NM_002961//S100A4//S100 calcium binding protein A4//1q21//6,275///NM_019554//	S100A4	0.0181,824	−3.73029
NM_001171171//CX3CR1//chemokine (C-X3-C motif) receptor 1//3p21.3//1,524///NM_	CX3CR1	0.0334,212	−3.35191
NM_001002273//FCGR2B//Fc fragment of IgG, low affinity IIb, receptor (CD32)//1q23	FCGR2B	0.0200,532	−3.33679
NM_001304428//GAPT//GRB2-binding adaptor protein, transmembrane//5q11.2//202,309	GAPT	0.032369	−3.33522
NM_178,129//P2RY8//purinergic receptor P2Y, G-protein coupled, 8//Xp22.33; Yp11.3	P2RY8	0.044621	−3.29474
NM_001024847//TGFBR2//transforming growth factor beta receptor II//3p22//7,048/	TGFBR2	0.0265,183	−2.41692
NM_001206729//AKT3//v-akt murine thymoma viral oncogene homolog 3//1q44//10000	AKT3	0.00369,053	−2.15279
NM_003656//CAMK1//calcium/calmodulin-dependent protein kinase I//3p25.3//8,536/	CAMK1	0.0153,531	−2.01817
NM_000395//CSF2RB//colony stimulating factor 2 receptor, beta, low-affinity (granulocyte-macrophage)	CSF2RB	0.0052095	−2.24987

### Ingenuity Pathway Analysis

**FIGURE 7:**
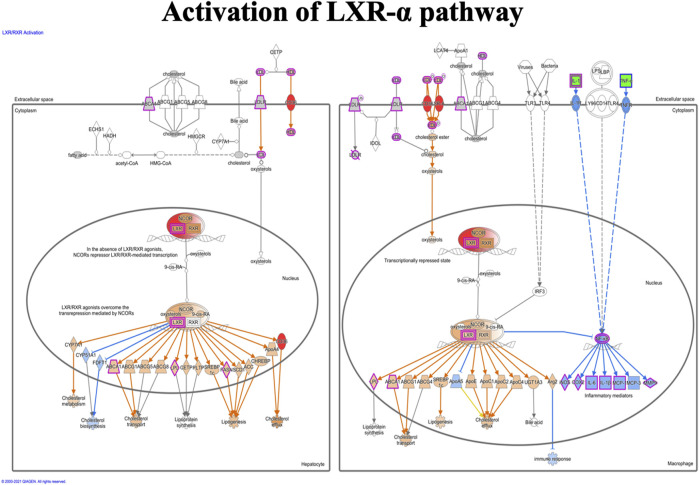
Activation of the LXR-α pathway in the macrophage. The LXR-α pathway shows the predicted effect of the detected seven molecules (APOC1, APOE, C9, CD36, IL1RAP, SR-A, and NR1H3 (LXR-α).

**FIGURE 8:**
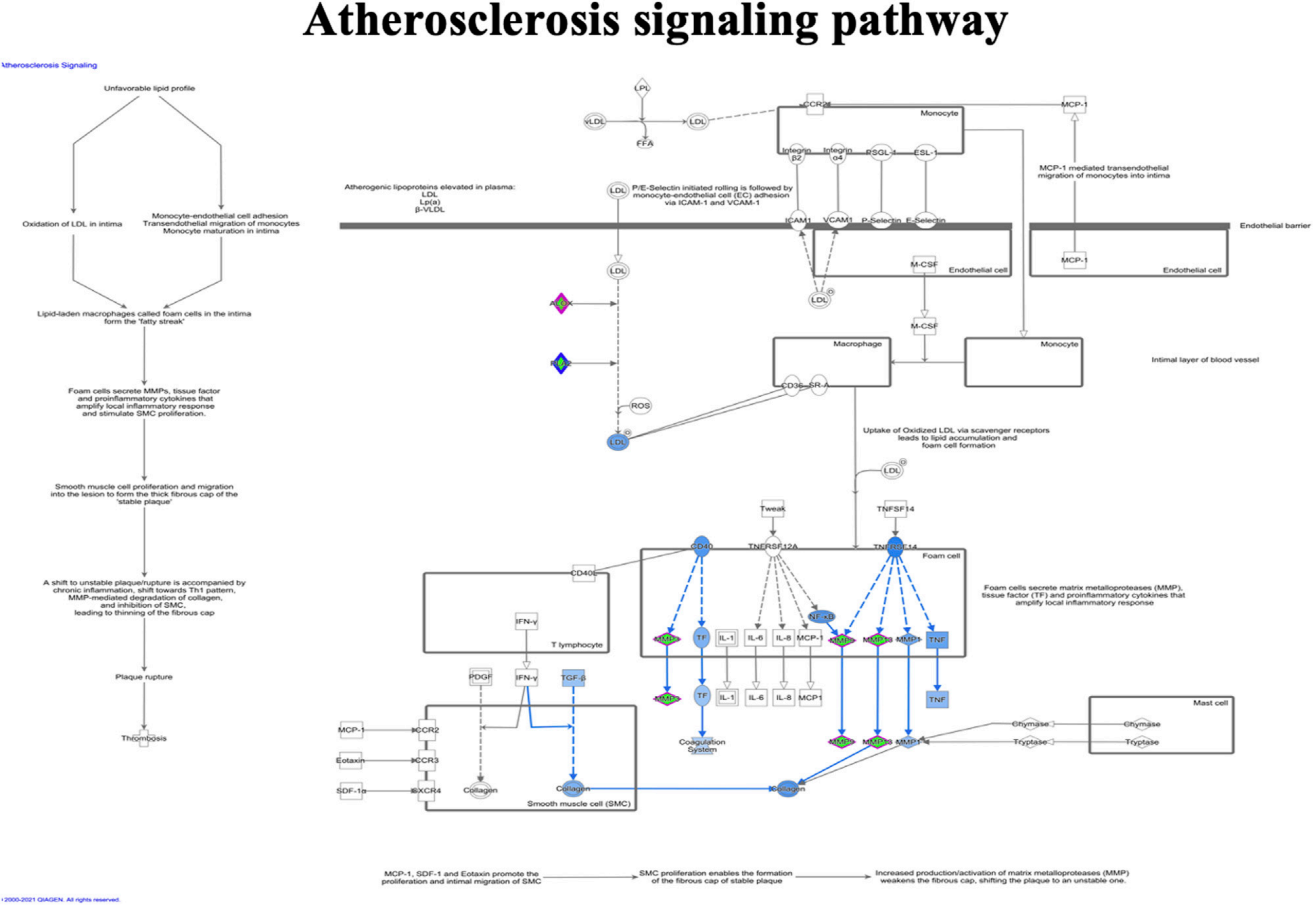
Ingenuity pathway analysis of genes/molecules linked to the atherosclerosis signaling pathway regulated by quercetin.

**FIGURE 9:**
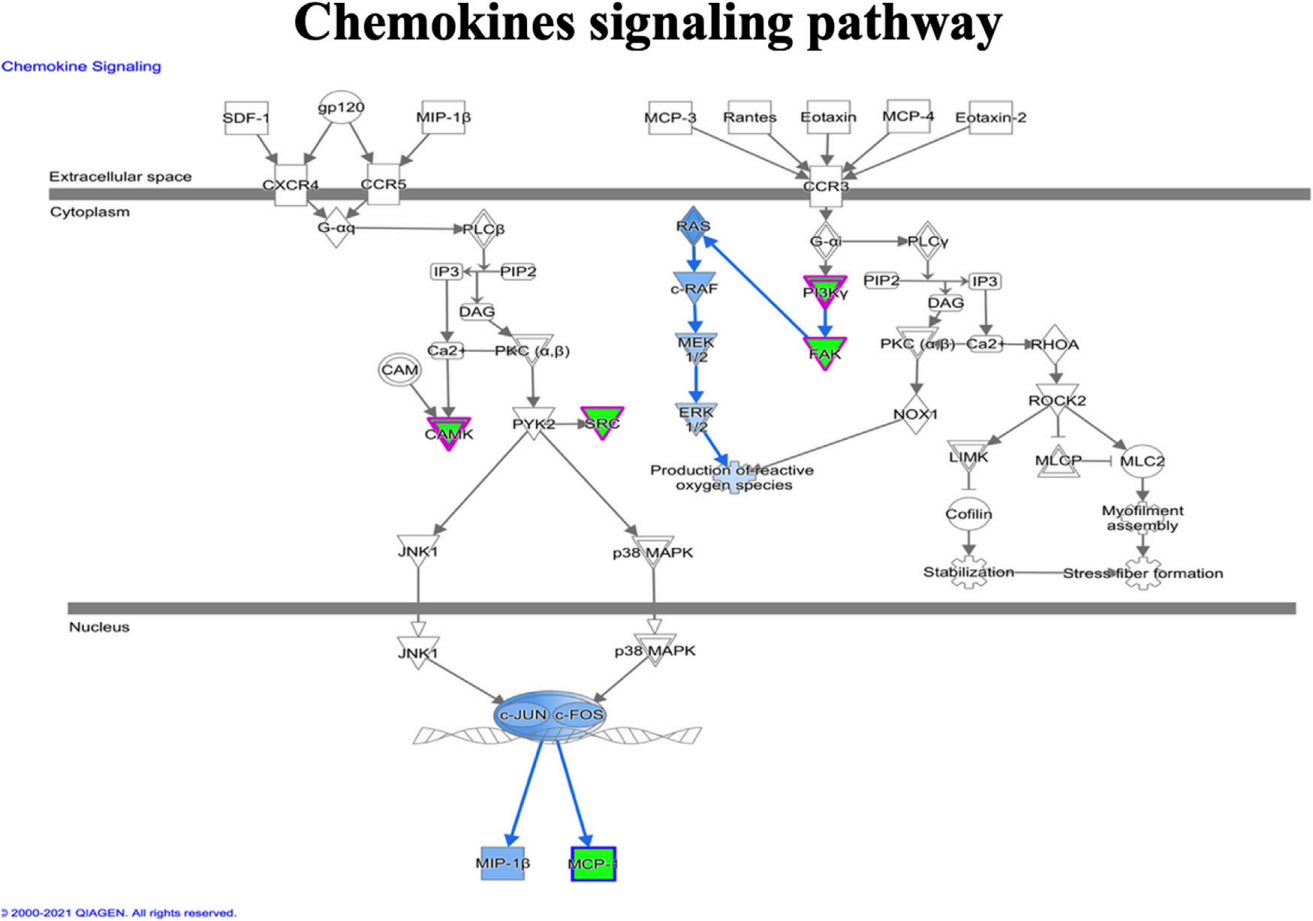
Ingenuity pathway analysis genes/molecules linked to chemokines signaling regulated by quercetin.

**FIGURE 10:**
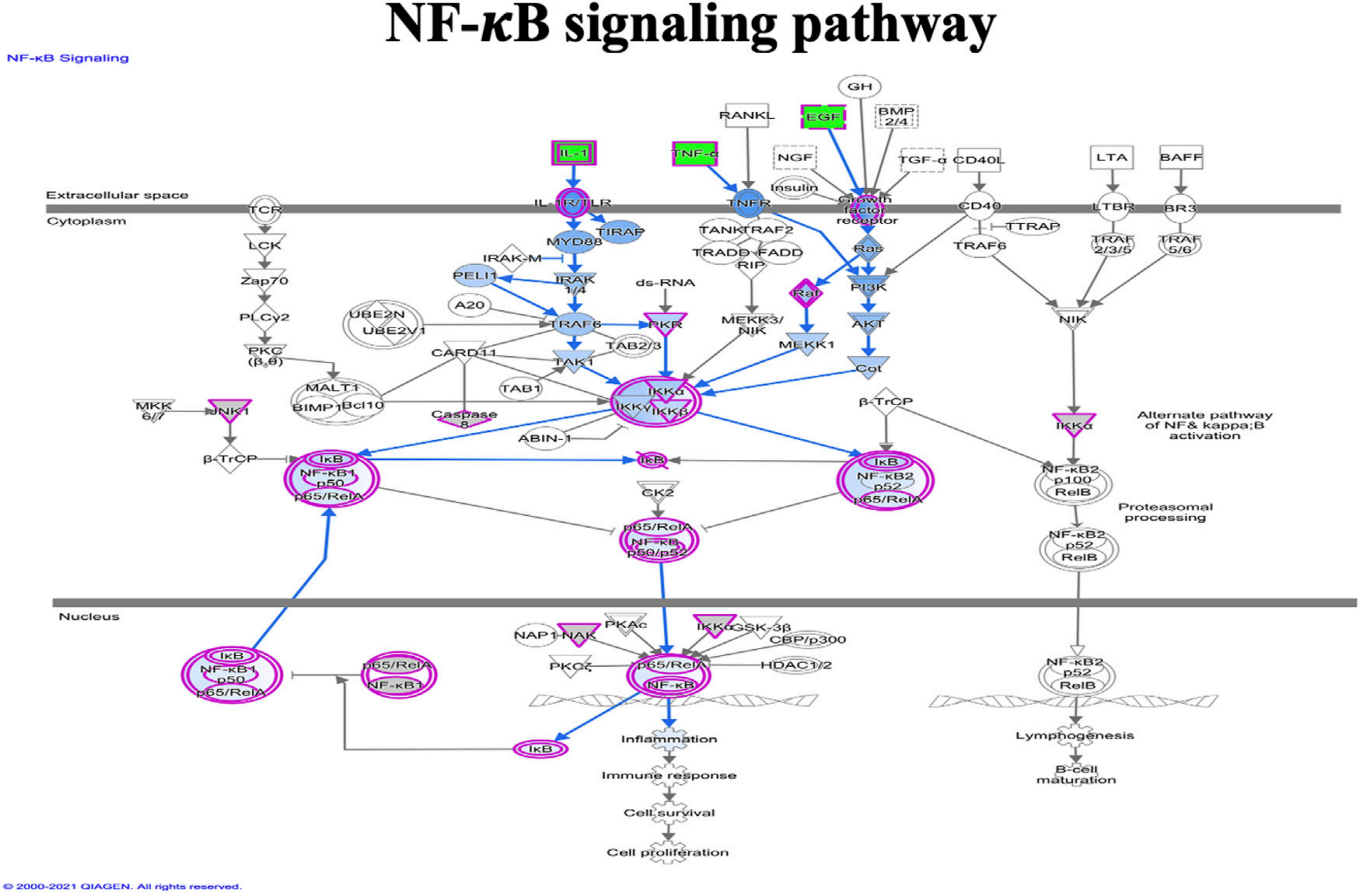
Ingenuity pathway analysis of genes/molecules linked to NF-κB signaling regulated by quercetin.

IPA analysis of the top 295 differentially expressed genes (DEGs) (data not shown) revealed canonical pathways, including molecules related to the LXR/RXR pathway (Figure 7), atherosclerosis signaling (Figure 8), chemokine signaling (Figure 9), and NF-κB signaling (Figure 10). The IPA analysis indicated that several DEGs were mainly involved in different pathways related to AS, inflammation, etc., as shown in Table 4.

**TABLE 4:** Molecular network analysis using IPA.

IPA	−Log (*p* value)	Ratio	z-score	Molecules
PI3K/AKT signaling	1.72	0.0405	1.342	AKT3, BCL2, CSF2RB, IL7R, ITGA2, ITGA3, and ITGB1
NF-κB signaling	0.556	0.0234	0	AKT3, PDGFRA, TGFBR2, and TLR1
LXR/RXR activation	2.55	0.0583	2.449	APOC1, APOE, C9, CD36, IL1RAP, MSR1, and NR1H3
GNRH signaling	0	0.0118	0	ADCY8 and CACNA1G
Chemokine signaling	0.47	0.026	0	CAMK1 and CXCR4
Atherosclerosis signaling	1.38	0.0413	0	APOC1, APOE, CD36, CXCR4, and MSR1

### Deciphering Genes Involved in Cholesterol Uptake and Efflux in THP-1 Macrophages Using Real-Time Polymerase Chain Reaction

**FIGURE 11:**
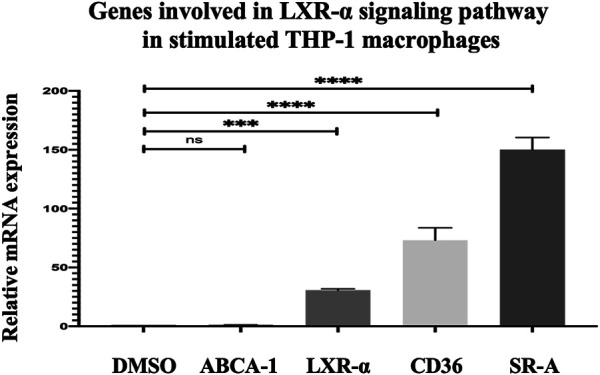
Effect of quercetin on ABCA-1 and LXR-α, CD36, and SR-A in stimulated THP-1 macrophages. The data are presented as the mean ± SD from three independent experiments. Statistical analysis was performed using a one-way ANOVA with Dunnett's test where *p < 0.05, **p < 0.01, ***p < 0.001, and ****p < 0.0001.

The genes involved in the LXR/RXR pathway, namely, ABCA-1, LXR-α, CD36, and SR-A in THP-1 macrophages after 24 h incubation with 15 μM quercetin, were evaluated to further understand the mechanism of quercetin in increasing cholesterol efflux and uptake. The LXR-α gene expression was increased by 29.68%-fold, and this increase was significantly significant (*p* < 0.0009) (Figure 11). The ABCA-1 which is the target gene of LXR-α did not show a significant increase (*p* = 0.9999) (Figure 11). Both CD36 and SR-A demonstrated statistically significant increases in gene expression by 72.16-fold (*p* < 0.0001) and 149.1%-fold (*p* < 0.0001), respectively, compared to the control (DMSO) (Figure 11).

## Discussion

Due to an increasingly observed role for inflammation in the development of AS disease, it has been suggested that the anti-inflammatory role of quercetin can mediate its protective effects against AS. IFN-γ expression has been documented to be highly expressed in AS lesion and to play a key function in disease progression (Li et al., 2010; Moss and Ramji, 2016).

Quercetin had no significant impact on ICAM-1 gene expression in THP-1 cells differentiated with PMA, which may be because the ICAM-1 expression at the basal level is very low, unlike the cells stimulated with IFN-γ cytokine (Pak et al., 2014). Our findings show that the production of MCP-1 mRNA by PMA was greatly inhibited by quercetin. This is consistent with previous studies that demonstrated inhibition of MCP-1 in the dsRNA-induced RAW 264.7 macrophage cell line (Moss et al., 2016) and LPS-induced RAW 264.7 macrophage cell line (Xue et al., 2017), respectively.

Our analysis was associated with *in vitro* (Sun et al., 2015; Cheng et al., 2019). It has been reported that THP-1 cells treated with IFN-γ greatly enhanced the gene expression of MCP-1 and ICAM-1, which is implicated in the process leading to the early stage of AS plaque formation (Harvey et al., 2007; Kurihara and Furue, 2013; Moss et al., 2016). Different stimuli such as IFN-γ, ox-LDL, and TNF-α have been used in human macrophage cell lines such as THP-1, umbilical vein endothelial cells (huvec), U937 cell line, and rat aortic smooth muscle cells (RASMCs) and J744.2 (Harvey et al., 2007; Chen et al., 2015; Bhaskar et al., 2016; Moss et al., 2016). They found that ICAM-1 and MCP-1 were remarkably overexpressed under IFN-γ stimuli and cytokines, such as IL-1β and ox-LDL. Our analysis was associated with experiments conducted *in vitro*, and the observed inhibition of ICAM-1 and MCP-1 with quercetin agreed with an earlier study where quercetin inhibited ox-LDL-stimulated ICAM-1. These adhesion molecules are overexpressed in EC dysfunction, indicating cardiovascular disease (Zhong et al., 2012; Jackson et al., 2018). In HUVECs, quercetin exhibited a potent anti-inflammatory effect by downregulating ICAM-1 mRNA gene expression (Bhaskar et al., 2016).

In addition, monocyte migration is involved in the early stages of AS induced by the physiological concentration of MCP-1. Our study showed that quercetin significantly inhibited the migration of monocytes. Monocytes that are recruited to the damaged EC site infiltrate into the atrial wall and differentiate into macrophages. Mediating this progression is the adhesion molecule ICAM-1 and the essential chemokine MCP-1 (Čejková et al., 2016). An earlier study also showed that these genes mediate the signaling pathways involved in the monocyte migration of monocytes (Li et al., 2010).

IFN-γ can downregulate gene-expression–related cholesterol efflux and decrease the efflux of cholesterol from foam cells (Voloshyna et al., 2014; Shao et al., 2018). We also noted that quercetin significantly increased cholesterol efflux, which is in line with an earlier study (Lu and Jia, 2016). Current research shows that quercetin has a significant effect on the expression of cholesterol efflux master regulators in human macrophages by increasing CD36 and SR-A. Previous studies have shown that the two essential SR, SR-A and CD36 receptors, which are highly expressed in macrophages, control the uptake of modified LDL (Yamamoto et al., 2016; Li et al., 2017). Moore et al. established using both *in vivo* and *in vitro* studies that loss of SR-A or CD36 gene expression, which is involved in lipid uptake, does not impede the progression of atherosclerotic lesions (Moore et al., 2005). Lipid uptake by SRs can protect against the development of atherosclerotic lesions, which may be necessary to activate the LXR/RXR signaling pathway. An earlier *in silico* study noted that this flavonol is involved in CYP1B1 modulation. CYP1B1 is a superfamily of enzymes that belongs to the cytochrome P450 (Elfaki et al., 2018) and catalyzes the metabolism of drugs and other substances. Endogenous substrates of CYPs include lipid mediators such as eicosanoids, estradiol, arachidonic acids, and cholesterol (Li et al., 2017). In animal model studies, CYP1B1 was found to play a crucial role in the development of hyperlipidemia, atherosclerosis, and generation of reactive oxygen species (ROS) (Song et al., 2016; Elfaki et al., 2018). Our findings showed that CYP1B1 was inhibited by quercetin, which is similar to previous research using human cancer cell lines, including THP-1, HUVECs, and human aortic endothelial cells (HAECs) (Chaudhary and Willett, 2006; Li et al., 2017; Feng et al., 2019).

In an inflammatory response to the pathogenesis of AS (McLaren et al., 2011), LXRs play a central role in regulating important genes. Our findings showed that MCP-1 was inhibited at the baseline level and may be attributed to the activation of LXR expression.

Programmed cell death in macrophages plays an important role in various stages of AS (Yancey et al., 2011; Linton et al., 2016). In addition, cell proliferation and transformation mechanisms are associated with the apoptosis process (Romashkova and Makarov, 1999). PDGF is a growth factor that is involved in the inhibition of programmed cell death and stimulates cell survival (Romashkova and Makarov, 1999). Our IPA analysis indicated that the NF-κB pathway was involved in this signaling pathway, and this pathway may be critical in plaque stability and AS progression, as it is a potent stimulator of inflammation, apoptosis, and proliferation (Mehrhof et al., 2005; Son et al., 2012). We observed that quercetin induces PDGFRA and downregulates AKT3, and NF-κB is an antiapoptotic target that acts through the Ras/PI3K/Akt pathway (Mehrhof et al., 2005). The two independent mechanisms of cell death activated by oncogenic Ras are the antiapoptotic PI3K/Akt pathway and the proapoptotic Raf/MAPK pathway. Antiapoptotic NF-κB signaling can be activated by AKT, which indicates that quercetin may decrease PDGF signals through inhibition of PDGFR and block tyrosine kinase phosphorylation, thus decreasing signal transduction. However, different studies have shown that decreased levels of apoptotic macrophages are likely to increase lesions in early atherosclerotic lesions, but are correlated with plaque stability at the late stage (Gautier et al., 2009; Seimon and Tabas, 2009; Linton et al., 2016).

In addition, our IPA analysis showed that quercetin treatment activated the PI3K pathway. Huwait et al. reported that the LXR pathway is regulated by JNK/c-Jun/AP-1 (Huwait et al., 2011). In addition, they demonstrated for the first time the novel role of PI3K in the activation of nuclear receptors using ABCA-1 and APOE as model genes known to have an antiatherogenic effect.

## Conclusion

The present *in vitro* study shows that quercetin inhibits the overexpression of adhesion molecules ICAM-1 and chemokine molecule MCP-1 genes in both basal states following IFN-γ stimulation. This will help reduce the inflammatory effects and overcome endothelial dysfunction in AS. In addition, monocyte migration mediated by MCP-1 was inhibited by quercetin, which also helped in the prevention of AS. Achieving a critical balance between apoptosis and inflammation by targeting the involved signaling pathways is necessary. The natural flavonol quercetin has many useful properties such as anti-inflammation, monocyte inhibition, and increased cholesterol efflux; therefore, it may be used either alone or with existing therapeutics in AS. However, the present study did not include any *in vivo* animal studies, which would otherwise have substantially supported our *in vitro* findings. Further studies are needed to understand the underlying molecular mechanisms of the anti-inflammatory effects of quercetin and their role in monocyte migration, cholesterol efflux, and the LXR signaling pathways using both *in vitro* and *in vivo* disease models.

## Data Availability

The data generated in this study can be found in NCBI using accesion GSE160430.
